# Comparison of digital splints versus traditional splints for bruxism management: a systematic review

**DOI:** 10.1038/s41405-026-00438-9

**Published:** 2026-05-08

**Authors:** Ravinder S. Saini, Kanwalpreet Kaur, Seyed Ali Mosaddad, Artak Heboyan

**Affiliations:** 1 Department of Allied Dental Health Sciences, COAMS, King Khalid University, Abha, Saudi Arabia; 2 Rutgers School of Dental Medicine, Rutgers University, Newark, NJ USA; 3 Department of Research Analytics, Saveetha Dental College and Hospitals, Saveetha Institute of Medical and Technical Sciences, Saveetha University, Chennai, India; 4 Department of Prosthodontics, School of Dentistry, Shiraz University of Medical Sciences, Shiraz, Iran; 5 Department of Prosthodontics, Faculty of Stomatology, Yerevan State Medical University after Mkhitar Heratsi, Yerevan, Armenia

**Keywords:** Occlusion, Removable prosthodontics

## Abstract

**Background:**

The prevalence of bruxism in the adult population varies widely. Splints are commonly used to treat bruxism, protect teeth, and alleviate the adverse effects of grinding or clenching. This systematic review aimed to compare the effectiveness of digital versus traditional splints in managing bruxism, focusing in clinical outcomes such as symptom severity, pain reduction, bruxism event frequency, and muscle activity.

**Methods and material:**

A Systematic review was conducted following PRISMA guidelines. A comprehensive electronic database search was conducted across PubMed, Scopus, the Cochrane Library, Dimensions, and Google Scholar for scholarly journal articles comparing digital and traditional splints. Eligibility criteria included randomized controlled trials (RCTs) published in peer-reviewed journals that investigated the effectiveness of splints in bruxism management. The Cochrane Risk of Bias 2.0 was used to appraise the quality of the included randomized controlled trials.

**Results:**

The literature search yielded 2115 records, of which eight articles met the eligibility criteria. Additionally, the risk of bias assessment results indicated that most randomized controlled trials reported a low to moderate risk of bias. This study’s findings demonstrate the superiority of digital splints over traditional splints in managing bruxism. However, this difference was not statistically significant.

**Conclusions:**

Digital splints showed a tendency toward improved outcomes in managing bruxism compared to traditional splints, but the lack of statistical significance highlights the need for further research to confirm these findings.

## Introduction

In 2018, sleep bruxism (SB) was defined as a masticatory muscle activity (MMA) during sleep that is characterised as rhythmic (phasic) or non-rhythmic (tonic) and is not a movement disorder or a sleep disorder in otherwise healthy individuals. Awake bruxism (AB) is defined as a masticatory muscle activity during wakefulness that is characterised by repetitive or sustained tooth contact and/or by bracing or trusting of the mandible and is not a movement disorder in otherwise healthy individuals [1].

In the general population, bruxism prevalence rates show wide variability, with the highest estimates being ~8–31% in the adult population [1]. Sleep bruxism worsens during sleep but may also be accompanied by daytime parafunctional activity [1]. Although the exact etiology of bruxism remains unclear, several factors have been proposed, including stress, anxiety, sleep disorders, occlusal trauma (misalignment of teeth), and drugs.

Bruxism can be accompanied by various signs and symptoms, including tooth wear, jaw pain, headache, sensitivity, and inability to open or close the mouth [2]. It can also lead to be accompanied by the development of secondary pathologies, such as temporomandibular joint disorders (TMD) and tension-type headaches. Bruxism is a polymorphic behavior, traditionally diagnosed based on clinical evaluation (examination of the patient’s craniofacial structures, especially the dentition), self-report of bruxism-related symptoms, and use of wearable and/or easily removable tools such as dental occlusal splints and electromyography (EMG) [3, 4]. Physiological and behavioral approaches for treating bruxism include oral appliances, stress management, psychological interventions, physical therapy, and surgical procedures in more severe cases [5, 6].

Bruxism can also lead to impairment of the quality of life in terms of sleep disturbance, pain, discomfort, and dental problems caused by or contributing to the issue. These persistent symptoms can lead to diminished well-being. Treating bruxism can be associated with less sleep disturbance, reduced pain, and improved overall functioning [7, 8].

Splints are often used to treat bruxism. These splints can be categorized broadly into either traditional splints fabricated using alginate impressions and acrylic resins or digital splints designed using digital scanning and manufactured from high-strength materials such as medical-grade polymers [9, 10]. Oral appliances, occlusal splints, or bite guards commonly treat bruxism [11, 12]. Bruxism splints protect teeth from the harmful effects of clenching/grinding because they serve as a barrier, thereby preventing the upper and lower teeth from touching or grinding. Consequently, clenching/grinding splints reduce destructive wear on teeth, fractures to teeth, and other negative consequences to teeth. More importantly, splints work by sharing forces across the entire dental arch, thereby reducing the pressure on individual teeth.

The main difference between digital and traditional splints is not only the method of fabrication used but also the materials used for their construction. Traditional splints are typically made from heat-cured acrylic resins. In contrast, digital splints incorporate high-strength materials such as medical-grade polymers or high-tech ceramics, contributing to their better long-term performance [13]. Digital splints have emerged as a promising approach for managing bruxism. Studies have shown that stabilization splints (SS) and palatal splints (PS) can effectively reduce sleep bruxism (SB) activity when monitored using portable electromyographic (EMG) recording systems [14]. Innovative technologies, such as digital axiography, have been developed to aid in the planning and producing occlusal splints for bruxism management, showing the potential of digital tools to enhance treatment outcomes [15]. Furthermore, computer-assisted or digitally constructed occlusal splints have been proposed to be comparable or superior to conventional treatments for TMD and bruxism [16]. The use of digital workflows, such as 3Shape Digital Design Software, has been highlighted as effective in creating occlusal splints tailored to specific clinical situations, emphasizing the role of technology in optimizing treatment approaches [17]. Although digital splints offer promising benefits, it is essential to consider factors such as occlusal force prediction and the integration of intelligent systems for bruxism diagnosis and treatment [18].

Technological advancements have significantly transformed the creation and application of splints in dentistry, enhancing diagnosis and treatment [19, 20]. Digital imaging and CAD/CAM systems enhance the precision mapping of dental structures, enabling effective splint customization for various requirements [10]. Digital occlusal analysis tools provide detailed insights into bite patterns in bruxism diagnosis, which is essential for customized and effective splint therapy [21]. These technological advancements improve patient comfort, treatment efficiency, and the management of dental conditions through precisely engineered splints [22].

Two basic types of splints can be used depending on the severity of bruxism and its presentation; however, both are meant to protect the teeth from the crushing force of the clenched jaws. Soft splints consist of a thin material that cushions teeth and are fairly comfortable for people with relatively mild bruxism [23]. Hard splints, usually made of hard acrylic or similar materials, are more durable and protect teeth from excessive damage [24]. The latter can come in custom-made versions similar to soft splints. For optimal protection, obtaining a custom splint that fits accurately is advisable.

Importantly, splints are used for symptomatic rather than curative interventions for bruxism. This might explain why they are often used in combination with other approaches, such as stress management techniques or dental adjustments, occasionally facilitated by the same dental splint. Follow-up visits for the dentist’s physical control of the situation are crucial to ensure that the splint remains effective and if changes are required [25].

Digital splints (digital occlusal splints or digital bite guards) can help reduce jaw pain, headaches, and tooth damage [26]. They also treat bruxism, also known as sleep bruxism or tooth grinding. They provide a precise fit, made possible by digital impressions, computer-aided design, and manufacturing (CAD/CAM) technology [27–29].

Moreover, digital splints are more resilient and longer-lasting than traditional splints [30]. Because they are produced from high-strength materials, such as medical-grade polymers or high-tech ceramics, they face a much lower risk of wear and tear, which means increased longevity and reduced replacement frequency.

Moreover, digital splints can provide real-time spectral and analytical feedback [31]. Some digital splints have sensors that measure the bite forces individuals experience from bruxism [31, 32]. This information can be saved digitally, which can later be used to record and analyze changes in biting patterns. According to the results, it can be adjusted for treatment provided by a healthcare professional. Furthermore, digital splints have the advantage of being more convenient than traditional splints. First, digital splints can be stored digitally, and there is no need to have a physical mold of the oral cavity, which simplifies the process of replacement or adjustments if necessary. Second, digital splints can be copied digitally if lost; no additional dentist appointments are required.

Despite these promising benefits, the comparative efficacy of digital versus traditional splints in managing bruxism remains an area of active research The aim of this systematic review was to compare the effectiveness of digital splints, fabricated using CAD/CAM or 3D printing technologies, versus traditional splints, made from alginate impressions, in managing bruxism. Effectiveness was evaluated based on clinical outcomes, including symptom severity, pain reduction, bruxism event frequency, and muscle activity, to inform clinical decision-making and optimize treatment approaches for bruxism.

## Methods and materials

The study was conducted according to the Cochrane Handbook for Systematic Reviews of Interventions and reported according to the Preferred Reporting Items for Systematic Reviews and Meta-Analyses (PRISMA) guidelines [33]. The protocol used for this systematic review was the registered International Platform of Registered Systematic Review and Meta-Analysis Protocols (INPLASY202420073).

### Study identification and selection

A preliminary study was conducted to provide an overview of the basic concepts of splints in managing bruxism. In addition, this initial search aimed to identify keywords used in the database search. A comprehensive database search was conducted using PubMed, Scopus, ScienceDirect, the Cochrane Library, and Google Scholar. The search covered articles published from inception to 13^th^ November 2023. The keywords used in different databases to optimize the search results were: dental splints, occlusal splints, bite splints, mouthguards, night guards, bruxism, tooth grinding, and tooth clenching. The search strings for all databases are presented in Table 1, which details the keywords and queries used to optimize the identification of relevant studies.

**Table 1:** Search strings of various databases.

Database	Search Strings
PubMed	(“Dental Splints”[All Fields] OR “Occlusal Splints”[All Fields] OR “Bite Splints”[All Fields] OR “Mouthguards”[All Fields] OR “Night Guards”[All Fields]) AND (“Bruxism”[All Fields] OR “Tooth Grinding”[All Fields] OR “Teeth Clenching”[All Fields])
Scopus	(“Dental Splints” OR “Occlusal Splints” OR “Bite Splints” OR “Mouthguards” OR “Night Guards”) AND (“Bruxism” OR “Tooth Grinding” OR “Teeth Clenching”)
ScienceDirect	(“Dental Splints” OR “Occlusal Splints” OR “Bite Splints” OR “Mouthguards” OR “Night Guards”) AND (“Bruxism” OR “Tooth Grinding” OR “Teeth Clenching”)
Cochrane Library	(“Dental Splints” OR “Occlusal Splints” OR “Bite Splints” OR “Mouthguards” OR “Night Guards”) AND (“Bruxism” OR “Tooth Grinding” OR “Teeth Clenching”)
Google Scholar	(“Dental Splints” OR “Occlusal Splints” OR “Bite Splints” OR “Mouthguards” OR “Night Guards”) AND (“Bruxism” OR “Tooth Grinding” OR “Teeth Clenching”)

Search strings were tailored to each database’s syntax where necessary, although the core keywords (“Dental Splints,” “Occlusal Splints,” “Bite Splints,” “Mouthguards,” “Night Guards,” “Bruxism,” “Tooth Grinding,” “Teeth Clenching”) remained consistent across platforms to ensure comprehensive coverage.

### Eligibility criteria

This study includes comparative research on the effectiveness of digital and traditional splints in managing bruxism. Articles that fulfilled the modified PICOS criteria were selected [34].

## Inclusion criteria

Studies were included if they met the following criteria:

- Population (**P**): Patients with bruxism- Bruxism diagnosis was verified through clinical evaluation, self-reported symptoms, or electromyography (EMG) as specified in the included studies, with no specific subclassification (e.g., sleep or awake bruxism) unless reported by the studies.
- Intervention (**I**): Digitally designed and fabricated splints, including occlusal splints, bite splints, night guards, mandibular advancement splints, and surgical splints, which are manufactured using CAD/CAM or 3D printing technologies or digital design tools (e.g., digital axiography or 3Shape Digital Design Software). Splints placed on either the maxillary or mandibular jaw were included, as specified by the studies. Was the fabrication process and the special design taken into account (e.g. use of digital axiography or 3Shape Digital Design Software) Were the splints placed in the upper or lower jaw or were both variants allowed
- Comparison (**C**): Traditionally fabricated splints are constructed manually using dental casts derived from alginate impressions, designed specifically for bruxism management, such as occlusal splints or bite guards.
- Primary outcomes (**O**): Efficacy, defined as the reduction in bruxism symptoms (e.g., symptom severity, pain, bruxism events). Secondary outcomes, such as patient compliance, acceptance, or muscle activity, were reported if included in the studies.
- Study Design (**S**): Randomized controlled trials. Clinical, observational, experimental, reviews, meta-analyses, case studies, non-comparative, and opinion studies were excluded.

## Exclusion criteria

- Splints designed for purposes other than bruxism management.
- Studies focusing solely on splint material composition without discussing digital vs. traditional fabrication.
- Non-clinical studies, such as in-vitro studies, finite element analysis, or mechanical testing studies without patient data.
- Opinion pieces, narrative reviews, or conference abstracts.

### Study selection

The search results were exported to Zotero Reference Citation Manager software version 6.0.36. Two reviewers (M.A.K and S.A.Q) selected the studies by screening their titles and abstracts. Potential articles were then searched for full-text retrieval. The retrieved articles were assessed for eligibility according to the mentioned pre-specified eligibility criteria. The first reviewer (M.K) selected the studies, after which the second reviewer (S.A) verified the eligibility of the chosen research articles against the eligibility criteria. The intervention of a third independent reviewer (R.S.S) harmonized any differing opinions of the two reviewers. A secondary search was conducted using the reference lists of the included studies.

### Data extraction

One reviewer (V.G) systematically extracted data from the selected studies, after which a second reviewer (M.A.K) cross-checked the data extraction results to verify accuracy and consistency. The extracted data were presented in a predesigned study descriptor table using Microsoft Excel Version 2021. The extracted data included the author, study design, sample size, mean age of the sample, study objectives, and study findings, as shown in Table 2.

**Table 2:** Detailed Characteristics of the considered studies [36, 43, 49–54].

Author	Study Design	Sample Size	Mean Age	Study Objectives	Digital Splints	Traditional splints	Findings
(Amin et al., 2016) [49]	Randomized clinical trial	45	18–65 (Age range)	To assess the effectiveness of using hard, liquid, and flexible splints to treat myofascial pain dysfunction syndrome.	Liquid and soft oral splints	Hard splints	The type of splint did not affect the three groups’ overall performance. The methods are cost-effective, have minimal side effects, and have high patient compliance.
(Dalewski et al., 2021) [54]	Randomized clinical trial	30	24.8	To investigate the immediate effect of two occlusal devices on pressure pain threshold values in patients diagnosed with bruxism.	Occlusal appliance by Okeson	Bimaxillary splint	The pain factors among the two groups were similar.
(Gu et al., 2015) [37]	Randomized controlled trial	24	25.65	To compare the effectiveness of biofeedback therapy to that of Occlusal splint therapy.	Biofeedback splint	Occlusal therapy	Biofeedback therapy provided better results than Occlusal therapy.
(Singh et al., 2015) [52]	Randomized trial	28	34.72	To investigate the effectiveness of MAD and maxillary occlusal splint on sleep quality and bruxism in patients.	Mandibular advancement device	Maxillary occlusal splint	MAD and MOS reduced sleep bruxism and improved sleep quality.
(Bergmann et al., 2020) [43]	Randomized controlled trial	41	37.6 ± 11 (Full-occlusion biofeedback (BFB) splint) and 41.3 ± 14.2 (Adjusted occlusal splint (AOS))	To compare treatment outcomes on sleep bruxism using Full-occlusion biofeedback (BFB) splint and adjusted occlusal splint (AOS).	Full-occlusion biofeedback (BFB) splint	Adjusted occlusal splint (AOS)	A full-occlusion biofeedback (BFB) splint is more effective than an adjusted occlusal splint (AOS) in reducing sleep bruxism and pain.
(Dalewski et al., 2015) [50]	Randomized controlled trial	30	26.63	To compare the efficacy of modified nociceptive trigeminal inhibition splints and Occlusal splints in bruxism management, electromyography activity levels during postural activity, and superficial temporal and masseter muscles maximum voluntary contraction.	Modified nociceptive trigeminal inhibition splints	Occlusal splint	The effects of the splints used on muscle activity were insignificant.

### Methodological quality assessment

One reviewer (A.O) used the Cochrane Risk of Bias Assessment tool (RoB 2.0) to assess the bias of the included clinical, randomized controlled trials and experimental studies [35].

### Data analysis and synthesis

The extracted data were thematically analyzed and reported according to the effectiveness of the splints [36]. Quantitative data were synthesized and organized according to the outcomes using Microsoft Excel version 2021.

## Results

### Study selection

**Fig. 1:**
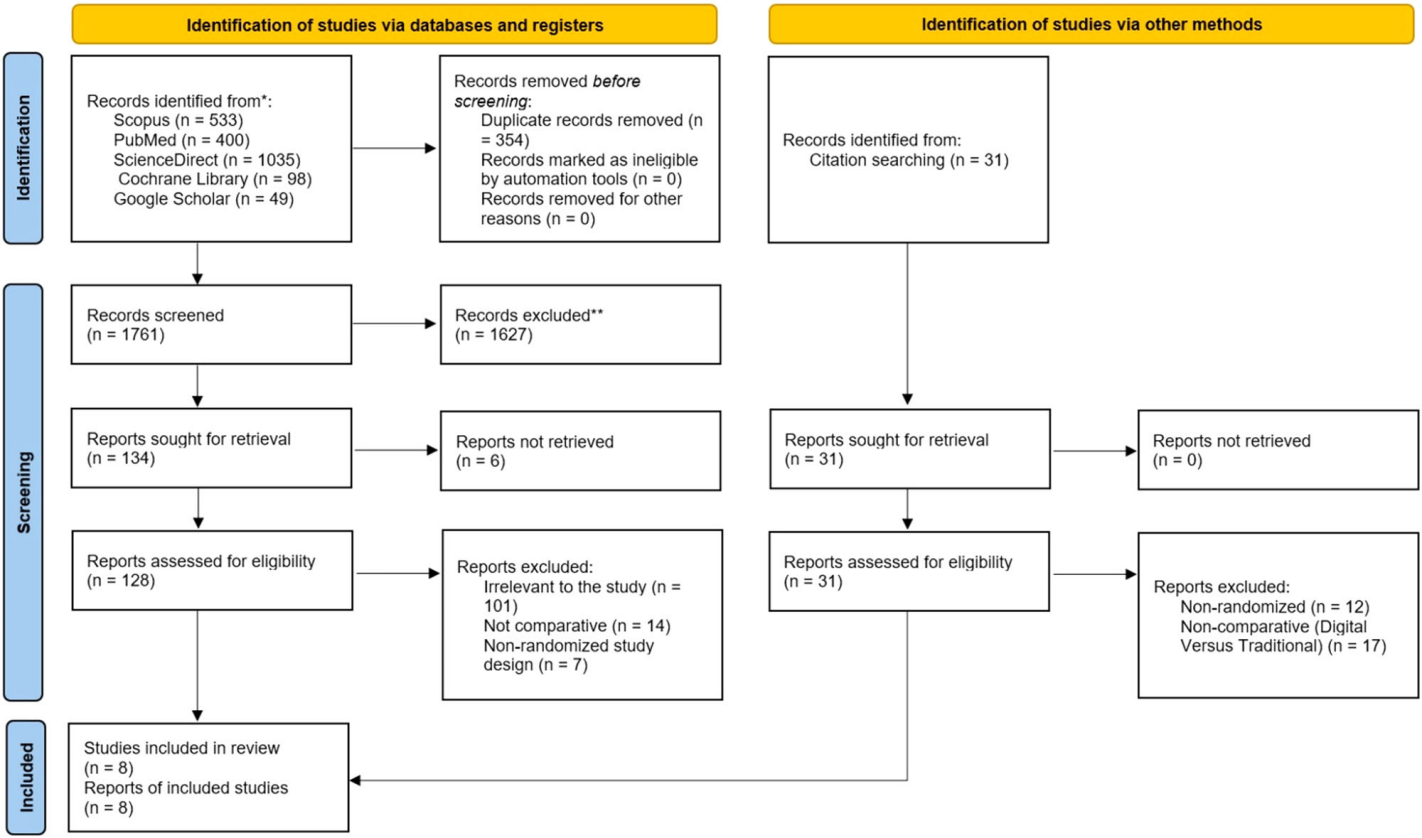
Study selection process for the systematic review. PRISMA 2020 flow diagram comparing digitally fabricated or digitally assisted splints with conventional splints for the management of bruxism. Records identified through electronic database searching and citation searching are shown separately. Number of duplicates removed, records screened, reports excluded and studies included in review are reported.

The literature search yielded 2115 articles, of which 354 duplicates were removed. Furthermore, 1627 articles were excluded following title and abstract screening. The remaining 134 articles were retrieved, and six studies that met the eligibility criteria were included. In addition, 31 articles were searched for citations, of which two were eligible and included in the study, to add the total to 8 eligible studies. The results are shown in Fig. 1.

### Methodological quality assessment

**Fig. 2:**
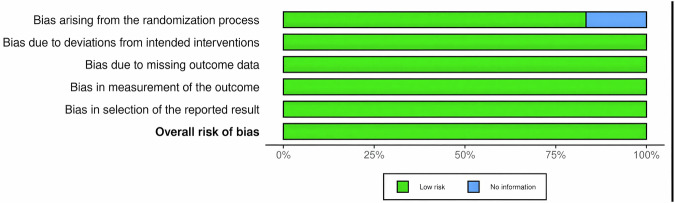
Summary of risk-of-bias judgments across included randomized studies. A Cochrane Risk of Bias 2.0 summary plot displays the percentage of studies judged to be in each category across domains for each risk of bias criterion. The categories for judgment are based on five domains of bias. They are bias arising from the randomization process, bias due to deviations from intended interventions, bias due to missing outcome data, bias in measurement of the outcome, and bias in selection of the reported result. Criteria shaded in green were assessed at low risk of bias. Those in blue mean there was no information provided.

**Fig. 3:**
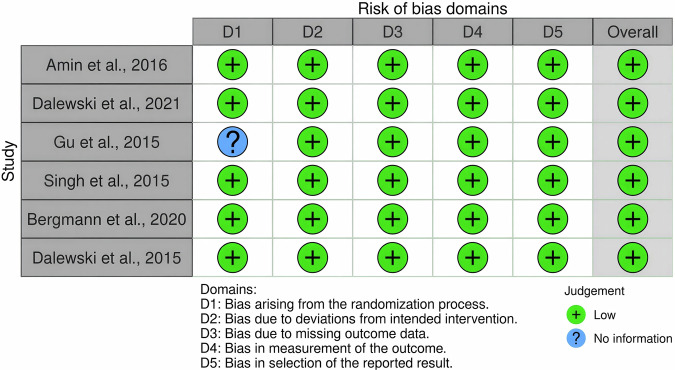
Study-level risk-of-bias assessment for included randomized studies. Traffic-light plot of the Cochrane Risk of Bias 2.0 assessments made for each randomized study included in the review. Included are judgments for each of five domains that contribute to risk of bias (D1 through D5) as well as the overall risk-of-bias judgment. Bias due to D1 refers to the randomization process; D2 to deviations from intended interventions; D3 to missing outcome data; D4 to measurement of the outcome; and D5 to selection of the reported result. A green circle with a plus sign means low risk of bias. A blue circle with a question mark means that there was no information.

The methodological quality of the included randomized controlled trials was assessed using the Cochrane Risk of Bias 2.0 tool, with results summarized in Figs. 2 and 3.

The “no information” ratings in several studies, as shown in Fig. 3, indicate missing details on randomization processes, allocation concealment, or outcome reporting. These gaps introduce uncertainty in the reliability of reported outcomes, potentially overestimating or underestimating the true effects of digital or traditional splints. For instance, incomplete reporting of blinding procedures may confound patient-reported outcomes like pain reduction or compliance. Approximately 60% of domains across studies were rated as low risk, 30% as some concerns, and 10% as “no information”, reflecting moderate overall methodological quality but highlighting gaps in reporting, particularly in older studies (e.g., van der Zaag et al., 2005), which may introduce selection or detection biases. This underscores the need for cautious interpretation of the findings and highlights the importance of standardized reporting in future trials.

### Data extraction

The researchers systematically extracted data from the included studies (Table 2).

### Results of individual studies

The extracted data were thematically analyzed according to the splint type, effectiveness, and compliance of the splints, as shown in Table 2.

### Effectiveness of splints

The efficacy of splints was assessed based on reductions in symptom severity, pain, and bruxism events. Studies reported that digital splints, such as biofeedback and CAD/CAM splints, showed greater reductions in symptom severity and pain compared to traditional splints, though not all differences were statistically significant [37, 38]. For example, Bergmann et al. (2020) found that full-occlusion biofeedback splints significantly reduced the frequency and duration of bruxism events compared to adjusted occlusal splints [37]. Similarly, Gu et al. (2015) reported better outcomes with biofeedback splints compared to traditional occlusal therapy [38].

Table 3 Quantitative data were analyzed to compare the effectiveness of digital and traditional splints. Effect sizes (Cohen’s d) and 95% confidence intervals were calculated to assess the magnitude and precision of differences in outcomes, including the number of bursts per hour, bruxism episode frequency, duration, and sleep quality metrics. These analyses provide insight into the clinical relevance of the observed differences. This study’s findings indicate that digital splints showed greater reductions in bruxism symptoms compared to traditional splints, though these differences were not statistically significant in most studies.

**Table 3:** Outcomes of the included studies.

Outcomes	Digital	Traditional	Effect size(Cohen’s d)	95% CI
(Gu et al. 2015) [38]				
Average total episodes of bruxism events during 8 hours of sleep	3.3 ± 1.4	11.4 ± 3.7	2.93	[1.95, 3.91]
Bruxism events duration	8.9 ± 2.7 s	14.0 ± 6.1 s	1.08	[0.41, 1.75]
(Singh et al., 2015) [53]				
Pittsburgh Sleep Quality Index	5.60 ± 0.51	6.40 ± 1.07	0.93	[0.29, 1.57]
Episodes of bruxism events with noise per hour	5.40 ± 2.45	5.80 ± 2.69	0.15	[-0.47, 0.77]
(Bergmann et al., 2020) [37]				
Effect of the splints on the number of bursts per hour	6.3 ± 0.42	3.58 ± 0.32	7.29	[5.32, 9.26]

## Discussion

This study evaluated a comparison between digital splints and traditional splints in the management of bruxism. This review examined digital splints fabricated using CAD/CAM technology and 3D printing methods as the intervention group compared these to traditional splints derived from conventional alginate impressions. The small sample sizes of the included studies likely contributed to the lack of statistical significance in some comparisons, highlighting the need for future studies with larger cohorts to confirm the observed trends in digital splint efficacy.

Findings from this study revealed that both digital and traditional splints are effective in reducing bruxism symptoms. However, digital splints demonstrated a greater overall reduction in symptom severity across most measured parameters, although not all differences reached statistical significance due to the relatively small sample sizes of the included studies. Research indicates that occlusal splints effectively treat bruxism by reducing the intensity of grinding forces, protecting teeth from damage, and alleviating possible orofacial pain associated with the condition [38]. These splints are considered a noninvasive and reversible treatment option for temporomandibular disorders and bruxism [39]. Studies have shown that patients using occlusal splints have a higher survival rate of dental restorations than those who do not use splints [40]. Additionally, occlusal splints are recommended as the first choice for bruxism treatment because of their effectiveness in relieving orofacial symptoms and reducing complications [41]. Technological advances have influenced the development and utilization of occlusal splints. Digital tools such as the digital axiograph and 3Shape Digital Design Software have been employed in the planning, design, and creation of occlusal splints to enhance precision and clinical outcomes [15]. Furthermore, the use of systems such as T-Scan Novus and BioEMG III allows for the adjustment and analysis of occlusal splints, thereby improving their fit and therapeutic effects [42].

Digital splints, fabricated using CAD/CAM or 3D printing technologies, demonstrated greater reductions in bruxism symptoms compared to traditional splints made from alginate impressions. These findings align with studies showing improved precision and fit with digital workflows, such as those using 3Shape Digital Design Software [18]. However, the lack of statistical significance in some studies suggests the need for further research with larger sample sizes. Many of these digital technologies have shown promise in personalized treatments for patients using digital technologies. In contrast, traditional occlusal and palatal splints have been widely used in bruxism despite limited or low-quality evidence supporting the efficacy of these splints. It is essential to conduct evidence-based and scientifically rigorous comparisons of the effectiveness of these two types of splints.

The findings of this study highlight the efficacy of digital splints over traditional splints in reducing bruxism symptoms, such as pain and bruxism events, though differences were not always statistically significant due to small sample sizes [37, 38]. These results suggest that digital splints, with their precise fit and advanced materials, may offer clinical advantages, warranting further investigation into their mechanisms and long-term effects.

These different splints were measured using different parameters, such as the decrease in the total number of bursts per hour [43] and the average number of bruxism events per 8 hours of sleep [37].

Finite element analysis studies have highlighted the biomechanical equilibrium created by occlusal splints, aiding in the treatment of bruxism by balancing physiological loading and stress generation [44]. Although occlusal splints have shown efficacy in managing bruxism-related symptoms, their effects on reducing electromyographic events were shown to be only transient [45].

Digital splints could be at the cutting edge of bruxism management, owing to the possibility of more personalized treatments that might improve patient comfort and relieve symptoms. However, research in this area is still needed to understand the exact mechanisms involved in their usefulness and to develop standards for implementing this fascinating tool in dental practice.

While digital splints offer many advantages, their application depends on various factors, such as costs, technological accessibility, and patient acceptance. The initial costs of digital splints may be higher due to advanced manufacturing techniques [46]. Additionally, patient acceptance may vary depending on comfort, adaptation time, and perceived effectiveness [47]. Future studies should evaluate these factors to enhance the implementation of digital splints in the management of bruxism. Additionally, future research should focus on conducting well-controlled randomized trials with standardized outcome measures to better assess the clinical significance of digital versus traditional splints in bruxism management.

## Study implications

The present study compared the effectiveness of traditional and digital splints and is critical for choosing splints to manage bruxism in patients. The important implications of this study are as follows:

1. Clinical Practice: The data will aid clinicians treating bruxism in deciding whether to use a conventional or digital splint.
2. Patient Counseling: The results may be helpful in patient counselling by increasing patients’ awareness regarding the perceived benefits of digital splints over conventional splints in the management of bruxism. The observed non-significant differences must be noted to appropriately manage patients’ expectations.
3. Product Development: The outcomes of this study could facilitate dental companies’ development of digital splint technology and mutually design new, creative ways of dealing with bruxism.
4. Research Priorities: More empirical work is likely needed before firm conclusions can be drawn, possibly making room for more groundbreaking, long-term, and varied research studies to enrich the evidence base.
5. Patient Outcomes: The findings can guide the selection of splint therapy by highlighting the potential of digital splints to enhance patient-reported outcomes, such as improved symptom relief (e.g., reduced pain and bruxism events), greater comfort due to precise fit, and enhanced quality of life, compared to traditional splints.

## Study limitations

The present study faced several limitations. The included studies had relatively small sample sizes, ranging from 13 to 45 participants, which limited the statistical power to detect significant differences between digital and traditional splints. Additionally, the heterogeneity in splint types and intervention methods across studies restricted the ability to perform a meta-analysis, affecting the generalizability and reliability of the findings. Future research should aim for standardized methodologies and larger sample sizes to improve comparability and strengthen the evidence base [48].

## Conclusions

This study compared the effectiveness of traditional and digital splints in managing bruxism. Digital splints showed a trend toward improved outcomes in managing bruxism compared to traditional splints, but the lack of statistical significance highlights the need for further research to confirm these findings.

## Data Availability

The data presented in this study are available on request from the corresponding author.
